# Scar extent evaluated by late gadolinium enhancement CMR: a powerful predictor of long term appropriate ICD therapy in patients with coronary artery disease

**DOI:** 10.1186/1532-429X-15-12

**Published:** 2013-01-19

**Authors:** Joachim Alexandre, Eric Saloux, Audrey Emmanuelle Dugué, Alain Lebon, Adrien Lemaitre, Vincent Roule, Fabien Labombarda, Nicole Provost, Sophie Gomes, Patrice Scanu, Paul Milliez

**Affiliations:** 1 Department of cardiology, CHU de Caen, Avenue de la Côte de Nacre, F-14000, Caen, France; 2 Department of biostatistics and clinical research, CHU de Caen, F-14000, Caen, France; 3 Université de Caen Basse-Normandie, Medical School, F-14000, Caen, France; 4 Department of radiology, CHU de Caen, F-14000, Caen, France

**Keywords:** Implantable cardioverter-defibrillator, Cardiovascular magnetic resonance, Scar tissue, Coronary artery disease, Sudden cardiac death, Ventricular arrhythmias

## Abstract

**Background:**

Coronary artery disease (CAD) patients are at risk for life-threatening ventricular arrhythmias (VA) related to scar tissue. Late gadolinium enhancement cardiovascular magnetic resonance (LGE-CMR) can accurately identify myocardial scar extent. It has been shown that scar extent, particularly scar transmurality, percent scar and scar mass, are associated with the occurrence of appropriate implantable cardioverter-defibrillator (ICD) therapy. However, quantification of transmurality extent has never been studied. The purpose of our study was to evaluate whether different methods quantifying scar transmurality, percent scar and scar mass (assessed with LGE-CMR) can predict appropriate ICD therapy in CAD patients with a long term follow-up period.

**Methods and results:**

We enrolled retrospectively 66 patients with chronic CAD referred for primary or secondary preventive ICD implantation and LGE-CMR before ICD implantation. Using LGE-CMR, scar extent was assessed by measuring scar mass, percent scar and transmural scar extent using four different methods. The median follow-up duration was 41.5 months (interquartile range 22–52). The endpoint was the occurrence of appropriate device therapy and occurred in 14 patients. Pre-ICD revascularization and transmural scar extent were significantly associated with the study endpoint but the latter was especially highly dependent on the method used. Patients with appropriate device therapy had also larger scar mass (29.6 ± 14.5 g vs 17.1 ± 8.8 g, p = 0.004), and larger percent scar (15.1 ± 8.2% vs 9.9 ± 5.6%, p = 0.03) than patients without appropriate device therapy. In multivariate analysis, scar extent variables remained significantly associated with the study end-point.

**Conclusions:**

In this study of CAD patients implanted for primary or secondary preventive ICD, pre-ICD revascularization and scar extent studied by LGE-CMR were significantly associated with appropriate device therapy and can identify a subgroup of CAD patients with an increased risk of life-threatening VA. Depending of the method used, transmural scar extent may vary significantly and needs further studies to obtain a validated and consensual study method.

## Background

Sudden cardiac death (SCD) is the most frequent cause of death in patients with coronary artery disease (CAD) [1]. Implantable cardioverter-defibrillator (ICD) implantation is a recognized beneficial therapy to prevent SCD related to ventricular arrhythmias (VA) in patients with low left ventricular ejection fraction (LVEF). However, identifying patients at high SCD risk remains a difficult challenge. Assessment of altered LVEF is still considered as the best discriminant factor of high risk SCD patients with CAD [2]. However its predictive accuracy is low [3]. Thus, many patients who receive ICD therapy in the light of current guidelines will never benefit from the device. Post hoc analysis of the MADIT II study population showed that only 35% of the patients who received an ICD for primary prevention receive appropriate device therapy during the first 3 years of follow-up [4]. Accordingly, better selection criteria for ICD implantation must be found.

Myocardial scar has been demonstrated as a substrate for malignant reentrant VA that may underlie SCD [5]. Late gadolinium enhancement cardiovascular magnetic resonance imaging (LGE-CMR) can accurately and reproducibly identify myocardial scar tissue and its extension [6,7]. The amount, as well as the transmural extent of myocardial scar tissue on LGE-CMR has been shown to predict overall mortality in patients with CAD independently of the LVEF [8,9] and thus may identify patients at risk of SCD. However, uniformity in the analysis of the LGE-CMR parameters is lacking and the different methods proposed to quantify the scar are not equivalent and seem poorly reproducible [10]. The most robust and reproducible parameters to quantify scar extent and to predict appropriate device therapy, appeared to be the scar transmurality and the amount of scar (percent scar and scar mass) [10-13].

The purpose of this study was to evaluate whether different methods quantifying scar transmurality, percent scar and scar mass (assessed with LGE-CMR) can predict appropriate ICD therapy in CAD patients with a long follow-up period.

## Methods

### Study population

The study was conducted in a retrospective observational manner in our cardiology department, at the Caen University Hospital (Normandy, France) during a period of 4 years (2006–2009), on 66 consecutive patients with CAD who had undergone LGE-CMR prior to primary or secondary ICD implantation. Ethical committee study procedures were in accordance with the Declaration of Helsinki. The study protocol did not require institutional review board approval since the study was performed retrospectively, only observational and patient data were anonymized and only patients from the Caen University Hospital Center (Caen, France) were included.

### CMR

All patients were scanned on a dedicated 1.5 T CMR scanner (Signa, GE Medical systems, Waukesha, WI) using a cardiac 5-element phased-array receiver coil. Images were acquired during breathholds of approximately 15 seconds using vector ECG gating. After initial localizer sequences, a stack of steady-state free precession cine images were acquired in the short axis plane from the level of the mitral valve annulus to the left ventricular (LV) apex. Contrast-enhanced images were acquired approximately 15 minutes after bolus injection of gadoterate meglumine, Dotarem® 0.15 mmol/kg (Guerbet, Aulnay-sous-Bois, France) using a standard 2-dimensional inversion recovery gradient-echo sequence [14].

### CMR image post-processing and data analysis

**Figure 1:**
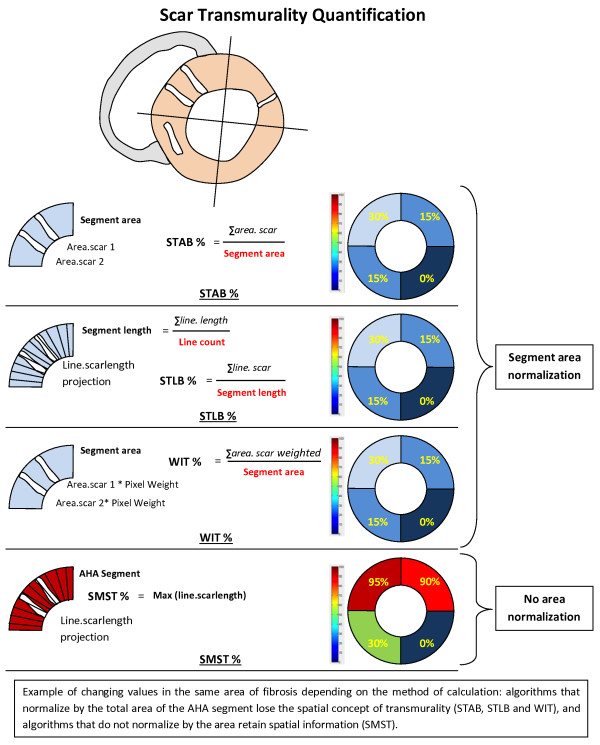
**Example of changing values in the same area of fibrosis depending on the method of calculation: algorithms that normalize by the total area of the AHA segmentation lose the spatial concept of transmurality (STAB, STLB and WIT), and algorithms that do not normalize by the area retain spatial information (SMST).** STAB indicates scar transmurality area based; STLB, scar transmurality line based; WIT, weighted infarct transmurality; SMST, spatial maximal transmurality.

All analyses were performed by an experienced cardiologist blinded to patient history using the freely available validated cardiovascular image analysis software package Segment v1.9 ( http://segment.heiberg.se) [15,16]. Short-axis cine images were used to measure end-diastolic volume, end-systolic volume, LV mass and LVEF by standard methods. Papillary muscles were regarded as part of the ventricular cavity. Scar analysis was performed using short axis LGE-CMR images. Endocardial and epicardial LV borders as scar tissue were semi-automatically delineated in each LV short-axis slice and then manually corrected. We worked with a binary approach to characterize scar tissue (normal myocardium vs. scar tissue). Three aspects of scar were quantified: the percent scar (percentage of the total LV volume), the scar mass and the transmural scar extent. The percent scar was calculated by summing the absolute amount of hyperenhanced tissue for all LV short-axis slices divided by the total amount of LV tissue. The scar mass was obtained by multiplying the percent scar by LV mass. For the transmural scar assessment, we used four different methods: 1) “scar transmurality area based” (STAB) based on the quantification of LV surface reached; 2) “scar transmurality line based” (STLB) based on the radial extent of late enhancement between the endocardial and epicardial borders; 3) “weighted infarct transmurality” (WIT) based on the LV segment mass reached weighted by pixel intensity to account for partial volume effect [17]. These first three methods are normalized by the segment AHA area and, consequently, lose their spatial information of scar. We therefore used the last method: 4) “spatial maximal scar transmurality” (SMST) that picks up maximal transmurality in the sector whereas the other three methods pick up different aspects of average transmurality. This method considers only the spatial distribution of scar, not its quantity compared with healthy tissue (Figure 1). The rationale behind using 4 different methods to quantify transmurality was to demonstrate that using the same term “transmurality” in different studies was not necessarily synonymous with comparable results. For the four methods, the transmural scar extent was split into quartiles (1–24%, 25–49%, 50–74% and 75–100%) [11], and the number of LV segments expressed in the standard American Heart Association 17-segment model [18]. For all methods, a presence of scar ≥ 75% was defined as transmural. All measurements were repeated in 18 patients by the same observer and by a second observer, blinded to the results of the first analysis, to assess intra-observer and inter-observer agreement.

### ICD implantation and details

All patients received an ICD according to international guidelines [19]. Some patients were eligible for cardiac resynchronization therapy (CRT) and received a combined CRT-D device as recommended [20]. All manufacturers were represented for either ICD (Lumax, Lexos, Lumos, Biotronik; Vitality 2, Teligen, Boston Scientific; Entrust, Virtuoso, Intrinsic, Medtronic Inc, Current, St Jude Medical) or CRT-D device (Contak, Contak renewal, Cognis, Boston Scientific [Natick, Mass, formerly Guidant Corp]; Lumax, Biotronik [Berlin, Germany]; Maximo, Concerto, InSync III and InSync Sentry, Medtronic Inc [Minneapolis, Minn]; Epic, Promote, Atlas, or Atlas II, St Jude Medical [St Paul, Minn]; Ovatio, Paradym, Sorin Group [Milan, Italy]).

Ventricular tachycardia (VT) zone was programmed from 171 ± 8/min to 217 ± 7/min with antitachycardia pacing therapy (ATP) (burst and/or ramp) then shock therapies. Arrhythmias faster than 217 ± 7/min were assigned to the ventricular fibrillation (VF) zone with maximal shocks as the first line therapy.

### Follow-up, events and end-point

Follow-up started at ICD implantation. All patients were followed 3 months after ICD implantation and then every 6 months. In our institution, some patients have a remote management system but were still followed as out patients every 6 months. Patients were instructed to contact the clinic after experiencing an ICD discharge for an additional visit. The median follow-up duration was 41.5 months (interquartile range 22–52). Appropriate ICD therapy was defined as ATP and/or shock therapy for VT or VF and was chosen as the study end-point. Appropriate arrhythmia detection and discrimination was confirmed by analysis of stored electrograms by two electrophysiologists blinded to the CMR analysis. When an appropriate ICD therapy occurred, acute reversible causes (particularly electrolyte disorders) and acute myocardial ischemia as a trigger for arrhythmic events were ruled out by electrocardiogram, standard blood examination and negative troponin levels. ICD therapy was classified as inappropriate when triggered by sinus or supraventricular tachycardia, T-wave oversensing, or electrode dysfunction.

### Statistics

Statistical analyses were performed on the R software version 2.14.0 (R Development Core Team, Vienna, Austria). Categorical variables were expressed as percentages (numbers) and compared using Fisher’s exact test between the two groups (receiving an appropriate ICD therapy or not). Continuous variables were presented as mean ± standard derivation and were compared between the two groups using a Student’s *t* test, or Mann–Whitney *U* test, if not normally distributed. The associations between the probability over time of receiving an appropriate ICD therapy and all clinical, electrocardiographic and CMR variables present in Tables 1 and 2 were first assessed in univariable Cox proportional hazards models (or by a log-rank test in case of a categorical variable for which the Cox model did not converge) but for the sake of clarity only significant variables (and LVEF and amiodarone share their clinical significance) are shown in Table 3. A multivariable model was then constructed with the most significant scar and clinical variables in the univariable analysis, respectively the scar mass and any previous pre-ICD revascularization as the covariable. We inserted only two covariables in the multivariate model due to the small number of patients receiving an appropriate ICD therapy (n = 14), and we used only one scar variable in the multivariate model because of the high collinearity between scar, percent scar and the different transmurality quantification method. The best model was defined by the log-likelihood test. Unadjusted and adjusted hazard ratios (HR) with their corresponding 95% confidence interval (CI) were reported. We therefore performed Receiver operator characteristic (ROC) analyses on significant predictors. In all analyses, a p value less than or equal to 0.05 was considered statistically significant.

**Table 1:** Baseline study population characteristics

	**Appropriate device therapy (n = 14)**	**No appropriate device therapy (n = 52)**	***p *value**
**Age, yrs**	60 ± 10	64 ± 11	0.17
**Male gender, n (%)**	14 (100)	50 (96)	0.46
**Previous MI, n (%)**	13 (93)	46 (88,5)	0.63
**Pre-ICD revascularization, n (%)**	3 (21,4)	38 (73,1)	< 0.001
**Previous PCI, n (%)**	3 (21.4)	26 (50)	0.056
**Previous CABG, n (%)**	0 (0)	14 (26.9)	0.029
**ICD indication, n (%)**			
**Primary prevention**	11 (78.6)	40 (76.9)	0.90
**Secondary prevention**	3 (21.4)	12 (23.1)	0.90
**NYHA functionnal class**	1.9 ± 0.7	2.0 ± 0.7	0.45
**Hypertension, n (%)**	3 (21.4)	25 (48.1)	0.07
**Diabetes, n (%)**	4 (28,6)	12 (23,1)	0.67
**Device type, n (%)**			
**ICD single chamber, n (%)**	11 (78.6)	30 (57.7)	0.15
**ICD dual chamber, n (%)**	1 (7.1)	8 (15.4)	0.42
**CRT-D, n (%)**	2 (14.3)	14 (26.9)	0.33
**ICD VT treatment zone lower setting (bpm)**	168 ± 7	171 ± 8	0.24
**ICD VF treatment zone lower setting (bpm)**	216 ± 8	217 ± 7	0.66
**Medication, n (%)**			
**Beta-blocker**	14 (100)	52 (100)	1.00
**ACE inhibitor or ATII antagonist**	14 (100)	50 (96.2)	0.46
**Statin**	13 (92.8)	49 (94.2)	0.85
**Amiodarone**	1 (7.1)	16 (30.8)	0.07
**Diuretics**	11 (78,7)	37 (71.1)	0.58
**Aldosterone blockers**	6 (42.9)	24 (46.1)	0.83
**Aspirin or clopidogrel**	14 (100)	51 (98.1)	0.60
**QRS duration, ms**	103 ± 24	117 ± 33	0.13
**Left bundle branch block, n (%)**	4 (28.6)	23 (44.2)	0.29
**Right bundle branch block, n (%)**	1 (7.1)	9 (17.3)	0.35
**Follow-up, months**	47 ± 18	41 ± 17	0.29

Continuous data are expressed as mean±SD and categorical data as n (%). *MI* indicates myocardial infarction; *PCI*, percutaneous coronary intervention; *CABG*, coronary artery bypass grafting; *ICD*, implantable cardioverter-defibrillator; *NYHA*, New York Heart Association; *CRT-D*, cardiac resynchronization therapy defibrillator; *ACE*, angiotensin-converting enzyme; *ATII*, angiotensin II.

**Table 2:** CMR variables

	**Appropriate device therapy (n = 14)**	**No appropriate device therapy (n = 52)**	***p *value**
**CMR-ICD delay, months**	1.6 ± 1.9	3.9 ± 6.5	0.20
**CMR haemodynamic data**			
**LVEF, **%	20.8 ± 9	24.1 ± 8	0.076
**LV EDV, ml**	275 ± 74	245 ± 50	0.073
**LV ESV, ml**	214 ± 47	196 ± 42	0.16
**LV mass, g**	205 ± 63	183 ± 36	0.09
**CMR-LGE**			
**Percent scar, **%	15.1 ± 8.2	9.9 ± 5.6	0.026
**Scar Mass, g**	29.6 ± 14.5	17.1 ± 8.8	< 0.005
**Transmural scar extent**			
**STAB method**			
**Total, n**	11.7 ± 3.8	10.4 ± 3.1	0.20
**≤24%, n**	5.6 ± 2.4	6.1 ± 2.4	0.45
**25-49%, n**	2.6 ± 1.7	2.3 ± 1.7	0.55
**50-74%, n**	2.0 ± 1.3	1.3 ± 1.3	0.09
**≥75%, n**	1.5 ± 1.9	0.7 ± 1.0	0.13
**STLB method**			
**Total, n**	11.9 ± 3.9	10.5 ± 3.1	0.20
**≤24%, n**	5.7 ± 2.1	6.2 ± 2.3	0.51
**25-49%, n**	2.7 ± 1.7	2.3 ± 1.7	0.49
**50-74%, n**	2.2 ± 1.3	1.4 ± 1.2	0.031
**≥75%, n**	1.3 ± 1.7	0.6 ± 0.9	0.22
**WIT method**			
**Total, n**	11.9 ± 3.9	10.5 ± 3.1	0.20
**≤24%, n**	7.9 ± 3.0	7.7 ± 2.4	0.99
**25-49%, n**	2.6 ± 2.1	2.1 ± 1.7	0.51
**50-74%, n**	1.3 ± 1.9	0.6 ± 0.8	0.53
**≥75%, n**	0.1 ± 0.3	0.1 ± 0.3	0.95
**SMST method**			
**Total, n**	12.4 ± 3.4	10.6 ± 3.1	0.062
**≤24%, n**	0.6 ± 0.8	0.7 ± 0.8	0.61
**25-49%, n**	1.3 ± 1.4	1.2 ± 1.5	0.72
**50-74%, n**	1.4 ± 1.6	1.7 ± 1.4	0.36
**≥75%, n**	9.1 ± 3.3	6.9 ± 3.5	0.03

Data are expressed as mean±SD. "n" indicates the number of LV segments. *LV* indicates left ventricular; *LV EDV, LV* end-diastolic volume; *LV ESV, LV* end-systolic volume; *LVEF*, left ventricular ejection fraction; *LV* mass, left ventricular mass; *CMR-LGE*, late gadolinium enhancement cardiac magnetic; *STAB*, scar transmurality area based; *STLB*, scar transmurality line based; *WIT*, weighed infarct transmurality; *SMST,* spatial maximal transmurality.

**Table 3:** Cox analysis of clinical characteristics and CMR variables for prediction of appropriate ICD therapy

	**Univariable analysis**		**Multivariable analysis**	
	**Hazard Ratio (95% CI)**	***p *value**	**Hazard Ratio (95% CI)**	***p *value**
**Clinical and ECG variables**				
Pre-ICD revascularization	0.102 (0.0226-0.461)	0.003	10.8 (2.1-53.6)	0.001
Amiodarone use	0.21 (0.0274-1.61)	0.13		
**CMR variables**				
LVEF	0.942 (0.869-1.02)	0.14		
Percent scar	1.08 (1.02-1.16)	0.014		
Scar mass	1.08 (1.04-1.12)	0.0001	3.15 (1.35-7.33)	< 0.001
Transmural scar extent				
STAB ≥75%	1.36 (1–1.83)	0.048		
STLB ≥ 50%	1.33 (1.04-1.69)	0.022		
SMST total	1.21 (1–1.45)	0.045		
SMST ≥75%	1.18 (0.993-1.39)	0.06		

*MI* indicates myocardial infarction; *LVEF*, left ventricular ejection fraction; *STAB,* scar transmurality area based; *STLB*, scar transmurality line based; *WIT*, weighed infarct transmurality; SMST, spatial maximal transmurality.

## Results

### Study population

During the study period, 66 patients with new ICD implants for CAD with a LGE-CMR prior to device implantation were included. Their baseline characteristics are shown in Table 1. Fifty-nine (89%) patients presented as ST-elevation MI, 39 patients (66%) received thrombolytic therapy and the other 20 patients (34%) were treated by primary percutaneous coronary intervention. Fifty-one patients (78%) presented with an initial 0 TIMI flow, 38 patients (58%) with a multivessel impairment and 56 patients (85%) were successfully treated by percutaneous coronary angioplasty. The median time frame between LGE-CMR and the respective coronary ischemic event was 4 months (interquartile range 3–6) and with ICD implantation was 3.4 ± 1.9 months. In all patients LGE-CMR was performed to guide the need for potential revascularization prior to ICD implantation including an assessment of myocardial viability. If necessary, a pre-ICD revascularization was performed before ICD placement (with a mean time frame of 1.7 ± 0.3 months). For the 41 patients who had pre-ICD revascularization, the LVEF did not significantly improve after revascularization and so did not modify the ICD indication.

### Follow-up and events

During a median follow-up of 41.5 months (interquartile range 22–52), study endpoint criteria was met in 14 patients (21%) and 10 patients died (15%). Non-cardiac death was reported in 2 patients (3%). Cardiac death occurred in 8 patients (12%): 7 patients (11%) died of end-stage heart failure and 1 patient (11%) died after heart transplant complications. In patients with appropriate ICD therapy, there was no significant difference between primary (n = 11) and secondary (n = 3) prevention indication (p = 0.90). Appropriate device therapy occurred 21 ± 20 months after ICD implantation. Eight of the 14 patients (57%) who presented the study endpoint were treated with ATP directly followed by shock or shock therapy only. The remaining 6 patients (43%) were successfully treated with ATP therapy only. The mean VT heart rate was 212 ± 32 bpm.

### CMR variables

CMR findings are listed in Table 2. All patients had evidence of scar tissue on contrast enhanced CMR. The mean distribution of the number of LV segments with transmural (≥ 75%) scar was: STAB: 0.8 ± 1.3; STLB: 0.7 ± 1.1; WIT: 0.08 ± 0.3; SMST: 7.36 ± 3.6, the mean percent scar was 11 ± 6.5%, the mean scar mass was 20 ± 11 g. As demonstrated in Table 2, depending on the method used, the transmurality quantification could differ significantly.

The percent scar, the scar mass, the number of LV segments with SMST ≥ 75% and the number of LV segments with STLB ≥ 50% (3.5 ± 2.4 vs 2.0 ± 1.6, p = 0.027) were significantly larger in patients who received appropriate ICD therapy compared with those who did not receive appropriate ICD therapy. For the STAB, WIT and SMST methods, the number of LV segment with a transmural extent ≥ 50% was not different between patients who did and did not met the study end-point.

All measurements were repeated in 18 randomized patients by the same observer and by a second observer, blinded to the results of the first analysis. The intraclass correlation coefficient for scar extent quantification was 0.91 for intra-observer agreement and 0.73 for inter-observer agreement (p < 0.001 for both), demonstrating high reproducibility. 

### Predictors of appropriate ICD therapy

**Figure 2:**
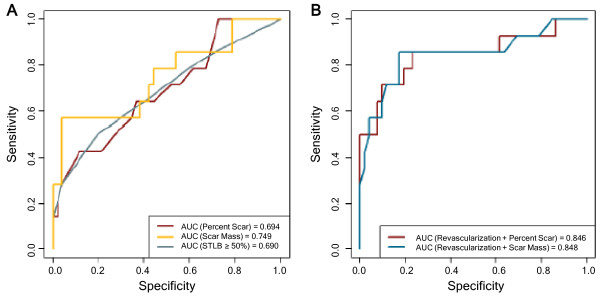
ROC curves of the univariate (panel A) and multivariate (panel B) Cox models.

As shown in Table 3 and Figure 2, univariate variables significantly associated with the study end-point were the number of transmural (≥ 75%) scar segments studied by the STAB method, number of segments with a scar extent ≥ 50% studied by the STLB method, total number of segments presenting scar studied by the SMST method, the percent scar, the scar mass, and any previous pre-ICD revascularization. Notably, LVEF (p = 0.14), QRS width (p = 0.14) and amiodarone use (p = 0.13) were not associated with the study end-point.

In our multivariate models, we could only retain the two most significant parameters due to our small study population. We took as scar variables the scar mass and the percent scar due to univariate analysis results. We included the notion of revascularization since it was the most strongly clinical parameter associated with appropriate device therapy (Table 3). Scar variables remained strongly associated with the occurrence of appropriate ICD therapy but the strongest association was with the scar mass (HR 3.15; 95% CI 1.35-7.33; p < 0.001 and HR 10.8; 95% CI 2.1-53.6; p = 0.001).

### CMR scar and Kaplan-Meier analysis

Median values of scar variables significantly associated with appropriate device therapy were used to individualize the risk of appropriate ICD therapy in this population. For the number of scar segments with transmural extent, patients were again separated into two groups based on the STLB method. We chose the STLB method because it presented the strongest association with the study end-point. Appropriate ICD therapy occurred in 11 of 30 patients with > 2 segments with a scar extent ≥ 50% compared with only 3 of 36 patients with ≤ 2 segments (p = 0.005). For the entire study population, the negative predictive value of ≤ 2 segments with a scar transmural extent ≥ 50% was 92% and sensitivity was 79% and the specificity 63%. For the scar mass (median value 20 g, 28 patients with a large scar mass > 20 g and 38 patients with a small scar mass ≤ 20 g), 10 patients (36%) with a large scar mass received appropriate ICD therapy compared with only 4 patients (11%) with a small scar mass (p = 0.01). The negative predictive value of a small scar mass was 90%, the sensitivity 71% and the specificity 65% for the entire study population. For the percent scar (median value 11%, 27 patients with a percent scar > 11% and 39 patients with a percent scar ≤ 11%), 9 patients (33%) with a large extent of scar received appropriate ICD therapy compared with only 5 patients (13%) with a small extent of scar (p = 0.04). The negative predictive value of a small extent of scar was 87% for the entire study population.

**Figure 3:**
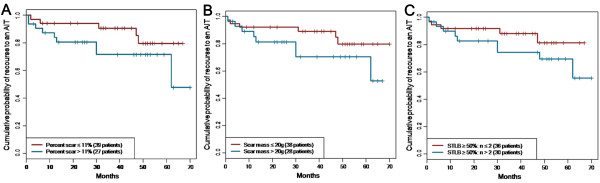
**Kaplan-Meier curve analysis in our population showing the difference in appropriate ICD therapy when patients are stratified according the median value of the percent scar (panel A), scar mass (panel B) and transmurality extent assessed with the scar transmurality line based extent ≥ 50% (panel C).** AIT indicates appropriate ICD therapy; STLB, scar transmurality line based, “n” the number of left ventricuar segments.

Kaplan-Meier survival curves for appropriate ICD therapy-free survival were calculated between patient groups stratified by median scar indices (scar mass, percent scar and number of transmural scar segments) (Figure 3).

## Discussion

Patients with chronic CAD are at risk of developing cardiovascular events and particularly VA, but different risk profiles exist. Traditional clinical indicators such as depressed LVEF, are still used to identify patients at risk of SCD [21], however these markers have low predictive values and many patients with an ICD will never benefit from the implantation [4]. Finding new indicators therefore remains a challenge. In our study, the only clinical parameter statistically associated with the study end-point is the notion of pre-ICD revascularization (Table 3). This finding is consistent with data from the literature. Indeed, the absence of pre-ICD revascularization was demonstrated as a risk factor for SCD and VA by Barsheshet *et al*[22]. In a multivariate analysis, they demonstrated that patients without prior revascularization had a 48% increased risk (p = 0.01) of VT/VF or death. This indicates that in addition to the scar tissue, myocardial region not revascularized before ICD implantation may predispose to recurrent ischemia or hibernation and could be an associated substrate for VA occurrence. They also showed that the association between pre-ICD revascularization and arrhythmic risk was similar among patients who underwent either coronary artery bypass graft (CABG) or percutaneous coronary intervention (PCI) as the last revascularization procedure prior to enrollment. In our study, there was a significant difference between PCI and CABG, but this result must be interpreted in the light of our relatively small cohort.

After a MI, scar tissue serves as an important substrate for VA, based on a re-entry phenomenon [23]. Consequently, assessment of scar extent by LGE-CMR could be useful for risk stratification of CAD patients. In this retrospective and observational study with long term follow-up, we confirmed that indices of LV scar extent, quantified by LGE-CMR, are associated with the occurrence of appropriate ICD therapy in CAD patients, independently of LVEF. These results are consistent with data in the literature. Scott *et al.* quantified myocardial scar in a 64 patients study with a mean follow-up of 19 ± 10 months [11]. The mean number of myocardial segments with transmural scar was 2.3 ± 2.1 and the mean percent scar was 14 ± 10%. These two criteria were significant predictors of appropriate device therapy (p = 0.001 and 0.02 respectively). These findings were also confirmed by Boyé *et al.* in 52 patients with chronic MI referred for primary preventive ICD implantation [24]. Infarct size was significantly larger in patients with appropriate device therapy or death (24 ± 8 g vs 16 ± 12 g, p = 0.02). One reason explaining that percent scar and scar mass are more significant than the transmural extent could be that in patients following MI, subsequent increases in infarct size correlate closely with the transmural extent of infarction, so increases in infarct size reflect increasing transmurality given the same area at risk [25].

**Figure 4:**
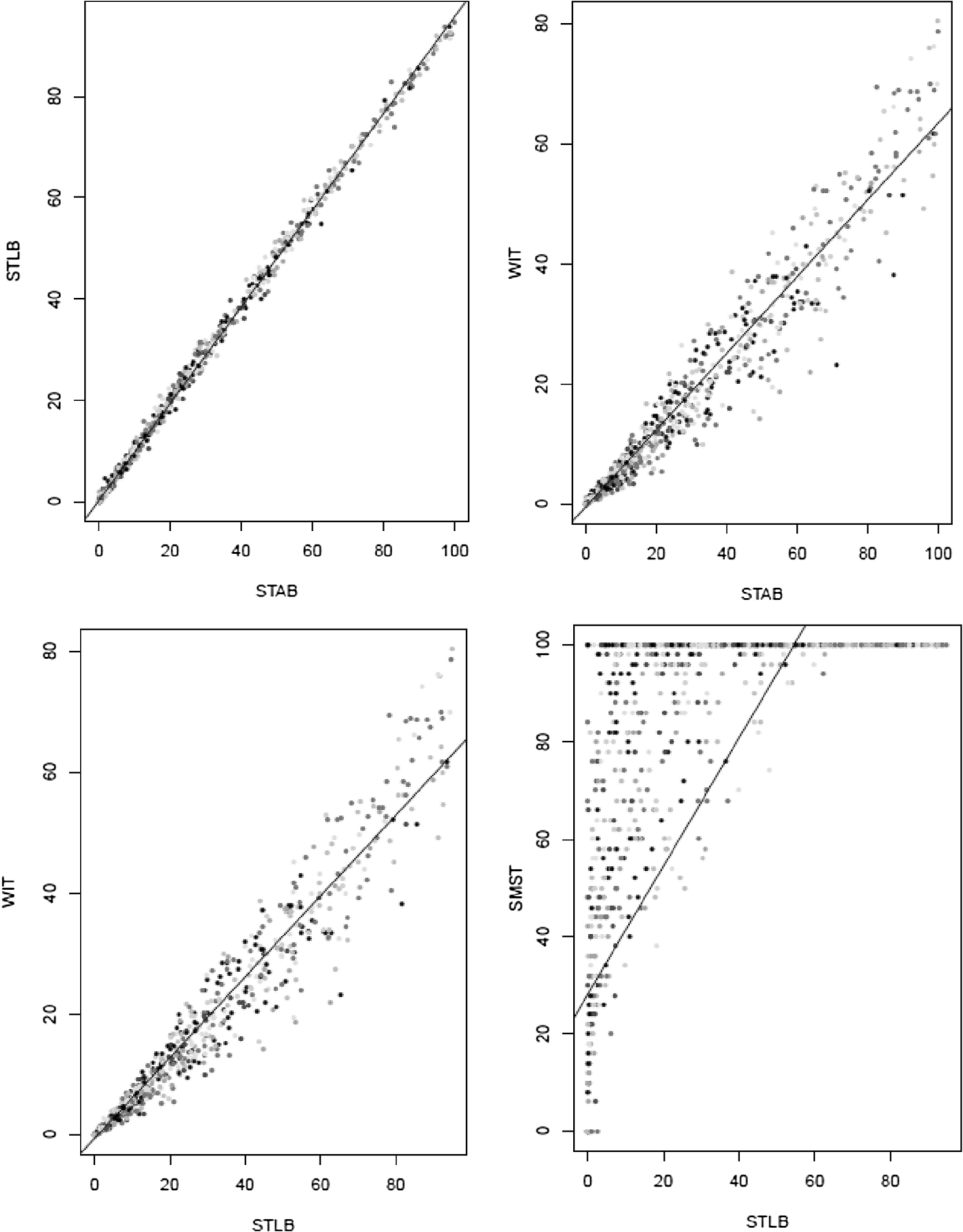
**Correlations between the four methods of scar transmurality quantification.** For each patient, there are 17 points corresponding to 17 left ventricular segments. STAB indicates scar transmurality area based; STLB, scar transmurality line based; WIT, weighed infarct transmurality; SMST, spatial maximal transmurality.

Quantitatively assessing infarct extent after MI has been a challenge for many years due to its important clinical implications. The concept of transmurality in humans is supported by coronary perfusion which is performed from the subepicardial to the subendocardial regions, resulting in a variable and heterogeneous extent in the myocardial wall in case of acute non-Q-wave MI and excess vulnerability of the subendocardial region to ischemia [26]. The goal during the following years was to develop methods to assess infarct size in order to prevent the development of myocardial injury following an acute MI. In this sense, special attention was paid to transmurality. Indeed, a large extent of transmural necrosis is known to induce deleterious remodeling [27] by loss of circular strain [28] that may itself be the cause of congestive heart failure. LGE-CMR is rapidly becoming the standard method due to its efficiency in detecting and distinguishing viable and nonviable myocardium [7,29]. To date, the transmurality concept is constantly used to assess infarct extension and severity but our understanding of transmurality is largely based on animal models [30,31] which may cause errors when transposing to humans. It should also be noted that currently, validation of methods to assess infarct size and transmurality after MI is limited [32] leading to mixed results for the same term "transmurality". This problem is well illustrated in our study. We observed a significant association between transmurality and appropriate ICD therapy but this is highly dependent on the method used (Table 2). The first 3 methods (STAB, STLB, WIT), by studying scar transmurality with the same approach based on normalization of the total area of the AHA segmentation regardless of the spatial concept of transmurality, are therefore more sensitive for scar mass than for transmurality. They tend to underestimate scar transmurality (most patients present a scar transmurality < 50%, Table 2). Moreover, as we can see in Figure 4, these 3 parameters are strongly correlated (R ≥ 0.959 for all 17 segments, p = 0). Conversely, the fourth method, by studying scar transmurality while considering spatial information, is more a reflection of the transmurality as the scar mass and therefore tends to detect considerable transmural damage (most of patients present a scar transmurality ≥ 75%, Table 2). In view of these results and the absence of consensus about transmurality quantification, the study of scar transmurality must remain a secondary endpoint in the CMR evaluation and should not participate to the decision of ICD implantation.

Recently, several studies abandoned the binary approach of scar quantification (scar tissue vs normal myocardium) and focused on the border zone around an infarct (peri-infarct zone) also determined by LGE-CMR. These studies have suggested that these parameters could be able to predict mortality, inducibility of VA, and the occurrence of appropriate ICD therapy [11,33-35]. However, these parameters seem to be time consuming, relatively operator-dependent, and are difficult to practice on a daily basis for the risk stratification of CAD patients [10]. In 55 patients and during a mean follow-up of 2 years, De Haan *et al.* evaluated previously validated methods of scar assessment by LGE-CMR in their ability to predict VA [10]. They suggested that quantification of total scar size with the binary approach (scar tissue vs. normal myocardium) is better and sufficient for SCD risk stratification of CAD patients. Moreover, in a recent experimental trial, Schuleri *et al.* studied the temporal evolution of the peri-infarct zone. They showed that the peri-infarct zone is dynamic and decreases over time and after a reperfused myocardial infarction [36].

### Limitation

This study was a unicenter observational trial, with a relatively small sample size and a small number of appropriate device therapies so the present conclusions require confirmation in larger cohort, prospective and multicenter studies. Moreover, from a pragmatic point of view, a cut-off value is needed to link scar extent to the identification of patients who are most likely to benefit from ICD implantation. Unfortunately, our study design prevents this. We have not been able to compare the extent of fibrosis in patients with primary and secondary prevention due to the small number of patients with secondary prevention. Furthermore, this trial included patients with CRT-D. Since biventricular pacing may also diminish the susceptibility to VA [37], this may have introduced a bias, although no significant difference was seen in the prevalence of CRT-D between the patients with and without appropriate device therapy. Finally, our sequences did not include coverage of the entire myocardium (just 1 short axis slice was missing), so we could possibly have missed small areas of scar.

## Conclusion

In this single-center study of patients with CAD and ICDs, we demonstrated a strong association between myocardial scar extent characterized by LGE-CMR with a binary approach (scar tissue vs. normal myocardium) and appropriate ICD therapy, independently of LVEF. Depending on the method used, transmural scar extent can predict appropriate ICD therapy in CAD patients but requires a validated and consensual study method. We hypothesize that this patient population could benefit from this study by using the scar extent to improve risk stratification strategies in CAD wanting to receive for an ICD.

## Abbreviations

CAD: Coronary artery disease;MI: Myocardial infarction;VA: Ventricular arrhythmia;CMR: Cardiovascular magnetic resonance;LGE-CMR: Late gadolinium enhancement cardiovascular magnetic resonance;ICD: Implantable cardioverter defibrillator;LVEF: Left ventricular ejection fraction;SCD: Sudden cardiac death;STAB: Scar transmurality area based;STLB: Scar transmurality line based;WIT: Weighed infarct transmurality;SMST: Spatial maximal scar transmurality;CRT: Cardiac resynchronization therapy;CRT-D: Cardiac resynchronization therapy defibrillator;VT: Ventricular tachycardia;VF: Ventricular fibrillation;ATP: Antitachycardia pacing therapy;ROC: Receiver operator characteristic;NYHA: New York health association;LV: Left ventricular;ESV: End-systolic volume;EDV: End-diastolic volume;CABG: Coronary artery bypass graft;PCI: Percutaneous coronary intervention;AHA: American heart association

## Competing interests

The authors declare that they have no competing interests.

## Authors' contributions

JA contributed to the study design, data collection, CMR sequence, data analysis and interpretation, and manuscript preparation. ES contributed to the study design, data analysis and interpretation, and manuscript preparation. AED contributed to data analysis and manuscript review. NP contributed to the CMR sequence. AL and AL contributed to the CMR sequence, data analysis, and manuscript review. VR, FL, PS, SG and PM contributed to the study design, data interpretation, and manuscript review. All authors approved the final version of the manuscript submitted.

## References

[B1] Zipes DP Wellens HJ Sudden cardiac death Circulation 1998 98 2334 2351 10.1161/01.CIR.98.21.2334 9826323

[B2] Epstein AE DiMarco JP Ellenbogen KA Estes NAM 3rd Freedman RA Gettes LS Gillinov AM Gregoratos G Hammill SC Hayes DL Hlatky MA Newby LK Page RL Schoenfeld MH Silka MJ Stevenson LW Sweeney MO Smith SC Jr Jacobs AK Adams CD Anderson JL Buller CE Creager MA Ettinger SM Faxon DP Halperin JL Hiratzka LF Hunt SA Krumholz HM Kushner FG ACC/AHA/HRS 2008 Guidelines for Device-Based Therapy of Cardiac Rhythm Abnormalities: a report of the American College of Cardiology/American Heart Association Task Force on Practice Guidelines (Writing Committee to Revise the ACC/AHA/NASPE 2002 Guideline Update for Implantation of Cardiac Pacemakers and Antiarrhythmia Devices): developed in collaboration with the American Association for Thoracic Surgery and Society of Thoracic Surgeons Circulation 2008 117 e350 e408 18483207 10.1161/CIRCUALTIONAHA.108.189742

[B3] Buxton AE Lee KL Hafley GE Pires LA Fisher JD Gold MR Josephson ME Lehmann MH Prystowsky EN Limitations of ejection fraction for prediction of sudden death risk in patients with coronary artery disease: lessons from the MUSTT study J Am Coll Cardiol 2007 50 1150 1157 10.1016/j.jacc.2007.04.095 17868806

[B4] Moss AJ Greenberg H Case RB Zareba W Hall WJ Brown MW Daubert JP McNitt S Andrews ML Elkin AD Long-term clinical course of patients after termination of ventricular tachyarrhythmia by an implanted defibrillator Circulation 2004 110 3760 3765 10.1161/01.CIR.0000150390.04704.B7 15583079

[B5] Haqqani HM Marchlinski FE Electrophysiologic substrate underlying postinfarction ventricular tachycardia: characterization and role in catheter ablation Heart Rhythm 2009 6 S70 S76 10.1016/j.hrthm.2009.04.023 19631910

[B6] Wu E Judd RM Vargas JD Klocke FJ Bonow RO Kim RJ Visualisation of presence, location, and transmural extent of healed Q-wave and non-Q-wave myocardial infarction Lancet 2001 357 21 28 10.1016/S0140-6736(00)03567-4 11197356

[B7] Kim RJ Fieno DS Parrish TB Harris K Chen EL Simonetti O Bundy J Finn JP Klocke FJ Judd RM Relationship of MRI delayed contrast enhancement to irreversible injury, infarct age, and contractile function Circulation 1999 100 1992 2002 10.1161/01.CIR.100.19.1992 10556226

[B8] Wu E Ortiz JT Tejedor P Lee DC Bucciarelli-Ducci C Kansal P Carr JC Holly TA Lloyd-Jones D Klocke FJ Bonow RO Infarct size by contrast enhanced cardiac magnetic resonance is a stronger predictor of outcomes than left ventricular ejection fraction or end-systolic volume index: prospective cohort study Heart 2008 94 730 736 10.1136/hrt.2007.122622 18070953

[B9] Kwon DH Halley CM Carrigan TP Zysek V Popovic ZB Setser R Schoenhagen P Starling RC Flamm SD Desai MY Extent of left ventricular scar predicts outcomes in ischemic cardiomyopathy patients with significantly reduced systolic function: a delayed hyperenhancement cardiac magnetic resonance study JACC Cardiovasc Imaging 2009 2 34 44 10.1016/j.jcmg.2008.09.010 19356530

[B10] de Haan S Meijers TA Knaapen P Beek AM van Rossum AC Allaart CP Scar size and characteristics assessed by CMR predict ventricular arrhythmias in ischaemic cardiomyopathy: comparison of previously validated models Heart 2011 97 1951 1956 10.1136/heartjnl-2011-300060 21917670

[B11] Scott PA Morgan JM Carroll N Murday DC Roberts PR Peebles CR Harden SP Curzen NP The extent of left ventricular scar quantified by late gadolinium enhancement MRI is associated with spontaneous ventricular arrhythmias in patients with coronary artery disease and implantable cardioverter-defibrillators Circ Arrhythm Electrophysiol 2011 4 324 330 10.1161/CIRCEP.110.959544 21493964

[B12] Roes SD Borleffs CJW van der Geest RJ Westenberg JJM Marsan NA Kaandorp TAM Reiber JHC Zeppenfeld K Lamb HJ de Roos A Schalij MJ Bax JJ Infarct tissue heterogeneity assessed with contrast-enhanced MRI predicts spontaneous ventricular arrhythmia in patients with ischemic cardiomyopathy and implantable cardioverter-defibrillator Circ Cardiovasc Imaging 2009 2 183 190 10.1161/CIRCIMAGING.108.826529 19808591

[B13] Yokota H Heidary S Katikireddy CK Nguyen P Pauly JM McConnell MV Yang PC Quantitative characterization of myocardial infarction by cardiovascular magnetic resonance predicts future cardiovascular events in patients with ischemic cardiomyopathy J Cardiovasc Magn Reson 2008 10 17 10.1186/1532-429X-10-17 18400089 PMC2322993

[B14] Hombach V Grebe O Merkle N Waldenmaier S Höher M Kochs M Wöhrle J Kestler HA Sequelae of acute myocardial infarction regarding cardiac structure and function and their prognostic significance as assessed by magnetic resonance imaging Eur Heart J 2005 26 549 557 15713695 10.1093/eurheartj/ehi147

[B15] Heiberg E Sjögren J Ugander M Carlsson M Engblom H Arheden H Design and validation of Segment–freely available software for cardiovascular image analysis BMC Med Imaging 2010 10 1 10.1186/1471-2342-10-1 20064248 PMC2822815

[B16] Heiberg E Engblom H Engvall J Hedström E Ugander M Arheden H Semi-automatic quantification of myocardial infarction from delayed contrast enhanced magnetic resonance imaging Scand Cardiovasc J 2005 39 267 275 10.1080/14017430500340543 16269396

[B17] Heiberg E Ugander M Engblom H Götberg M Olivecrona GK Erlinge D Arheden H Automated quantification of myocardial infarction from MR images by accounting for partial volume effects: animal, phantom, and human study Radiology 2008 246 2 581 588 18055873 10.1148/radiol.2461062164

[B18] Cerqueira MD Weissman NJ Dilsizian V Jacobs AK Kaul S Laskey WK Pennell DJ Rumberger JA Ryan T Verani MS Standardized myocardial segmentation and nomenclature for tomographic imaging of the heart. A statement for healthcare professionals from the Cardiac Imaging Committee of the Council on Clinical Cardiology of the American Heart Association Int J Cardiovasc Imaging 2002 18 539 542 12135124

[B19] Zipes DP Camm AJ Borggrefe M Buxton AE Chaitman B Fromer M Gregoratos G Klein G Moss AJ Myerburg RJ Priori SG Quinones MA Roden DM Silka MJ Tracy C Smith SC Jr Jacobs AK Adams CD Antman EM Anderson JL Hunt SA Halperin JL Nishimura R Ornato JP Page RL Riegel B Priori SG Blanc J-J Budaj A Camm AJ ACC/AHA/ESC 2006 guidelines for management of patients with ventricular arrhythmias and the prevention of sudden cardiac death: a report of the American College of Cardiology/American Heart Association Task Force and the European Society of Cardiology Committee for Practice Guidelines (Writing Committee to Develop Guidelines for Management of Patients With Ventricular Arrhythmias and the Prevention of Sudden Cardiac Death) J Am Coll Cardiol 2006 48 e247 e346 10.1016/j.jacc.2006.07.010 16949478

[B20] Strickberger SA Conti J Daoud EG Havranek E Mehra MR Piña IL Young J Patient selection for cardiac resynchronization therapy: from the Council on Clinical Cardiology Subcommittee on Electrocardiography and Arrhythmias and the Quality of Care and Outcomes Research Interdisciplinary Working Group, in collaboration with the Heart Rhythm Society Circulation 2005 111 2146 2150 10.1161/01.CIR.0000161276.09685.4A 15851622

[B21] Huikuri HV Castellanos A Myerburg RJ Sudden death due to cardiac arrhythmias N Engl J Med 2001 345 1473 1482 10.1056/NEJMra000650 11794197

[B22] Barsheshet A Goldenberg I Narins CR Moss AJ McNitt S Wang PJ Huang DT Hall WJ Zareba W Eldar M Guetta V Time dependence of life-threatening ventricular tachyarrhythmias after coronary revascularization in MADIT-CRT Heart Rhythm 2010 7 1421 1427 10.1016/j.hrthm.2010.07.005 20620231

[B23] de Bakker JM van Capelle FJ Janse MJ Wilde AA Coronel R Becker AE Dingemans KP van Hemel NM Hauer RN Reentry as a cause of ventricular tachycardia in patients with chronic ischemic heart disease: electrophysiologic and anatomic correlation Circulation 1988 77 589 606 10.1161/01.CIR.77.3.589 3342490

[B24] Boyé P Abdel-Aty H Zacharzowsky U Bohl S Schwenke C van der Geest RJ Dietz R Schirdewan A Schulz-Menger J Prediction of life-threatening arrhythmic events in patients with chronic myocardial infarction by contrast-enhanced CMR JACC Cardiovasc Imaging 2011 4 871 879 10.1016/j.jcmg.2011.04.014 21835379

[B25] Tarantini G Razzolini R Cacciavillani L Bilato C Sarais C Corbetti F Marra MP Napodano M Ramondo A Iliceto S Influence of transmurality, infarct size, and severe microvascular obstruction on left ventricular remodeling and function after primary coronary angioplasty Am J Cardiol 2006 98 1033 1040 10.1016/j.amjcard.2006.05.022 17027566

[B26] Ortiz-Pérez JT Meyers SN Lee DC Kansal P Klocke FJ Holly TA Davidson CJ Bonow RO Wu E Angiographic estimates of myocardium at risk during acute myocardial infarction: validation study using cardiac magnetic resonance imaging Eur Heart J 2007 28 1750 1758 10.1093/eurheartj/ehm212 17586811

[B27] Reimer KA Jennings RB The "wavefront phenomenom" of myocardial ischemic cell death. II. Transmural progression of necrosis within the framework of ischemic bed size (myocardium at risk) and collateral flow Lab Invest 1979 40 633 644 449273

[B28] Hung CL Verma A Uno H Shin SH Bourgoun M Hassanein AH McMurray JJ Velazquez EJ Kober L Pfeffer MA Solomon SD VALIANT investigators Longitudinal and circumferential strain rate, left ventricular remodeling, and prognosis after myocardial infarction J Am Coll Cardiol 2010 56 1812 1822 10.1016/j.jacc.2010.06.044 21087709

[B29] Ubachs JF Engblom H Erlinge D Jovinge S Hedström E Carlsson M Arheden H Cardiovascular magnetic resonance of the myocardium at risk in acute reperfused myocardial infarction: comparison of T2-weighted imaging versus the circumferential endocardial extent of late gadolinium enhancement with transmural projection J Cardiovasc Magn Reson 2010 29 12 18 10.1186/1532-429X-12-18 PMC2855565 20350309

[B30] Weisman HF Bush DE Mannisi JA Weisfeldt ML Healy B Cellular mechanisms of myocardial infarct expansion Circulation 1988 78 186 201 10.1161/01.CIR.78.1.186 2968197

[B31] Maroko PR Kjekshus JK Sobel BE Watanabe T Covell JW Ross J Braunwald E Jr Factors influencing infarct size following experimental coronary artery occlusions Circulation 1971 43 67 82 10.1161/01.CIR.43.1.67 5540853

[B32] Choi KM Kim RJ Gubernikoff G Vargas JD Parker M Judd RM Transmural extent of acute myocardial infarction predicts long-term improvement in contractile function Circulation 2001 104 1101 1107 10.1161/hc3501.096798 11535563

[B33] Yan AT Shayne AJ Brown KA Gupta SN Chan CW Luu TM Di Carli MF Reynolds HG Stevenson WG Kwong RY Characterization of the peri-infarct zone by contrast-enhanced cardiac magnetic resonance imaging is a powerful predictor of post-myocardial infarction mortality Circulation 2006 114 32 39 10.1161/CIRCULATIONAHA.106.613414 16801462

[B34] Schmidt A Azevedo CF Cheng A Gupta SN Bluemke DA Foo TK Gerstenblith G Weiss RG Marbán E Tomaselli GF Lima JAC Wu KC Infarct tissue heterogeneity by magnetic resonance imaging identifies enhanced cardiac arrhythmia susceptibility in patients with left ventricular dysfunction Circulation 2007 115 2006 2014 10.1161/CIRCULATIONAHA.106.653568 17389270 PMC2442229

[B35] Bernhardt P Stiller S Kottmair E Binner L Spiess J Grossmann G Rasche V Walcher D Hombach V Myocardial scar extent evaluated by cardiac magnetic resonance imaging in ICD patients: relationship to spontaneous VT during long-term follow-up Int J Cardiovasc Imaging 2011 27 893 900 10.1007/s10554-010-9726-9 20957518

[B36] Schuleri KH Centola M Evers KS Zviman A Evers R Lima JA Lardo AC Cardiovascular magnetic resonance characterization of peri-infarct zone remodeling following myocardial infarction J Cardiovasc Magn Reson 2012 14 24 10.1186/1532-429X-14-24 22510220 PMC3352163

[B37] Higgins SL Yong P Sheck D McDaniel M Bollinger F Vadecha M Desai S Meyer DB Biventricular pacing diminishes the need for implantable cardioverter defibrillator therapy. Ventak CHF Investigators J Am Coll Cardiol 2000 36 824 827 10.1016/S0735-1097(00)00795-6 10987605

